# Cell Doctrine

**Published:** 1879-02

**Authors:** 


*The Cell Doctrine—Its history and present state, for Students in Medicine and Dentistry. Also, a Copious Bibliography of the Subject.* By JAMES TYSON, M. D., Professor of General Pathological and Morbid Anatomy, in the University of Pennsylvania, &c., &c. Second edition revised, corrected and enlarged. Illustrated. Philadelphia: Lindsay & Blackiston, 1878. Buffalo: Theodore H. Butler.

This is a most carefully written and beautifully illustrated work upon the cell doctrine of the present day, containing a complete history of its origin, nature and growth, with an impartial review of the views and opinions represented by Prof. Huxley, Prof. Owen, Herbert Spencer, Grove, Tyndall, and many others, accurately detailing facts and conditions, by far the most interesting of all the objects known to physical science. We can hardly convey to our readers an idea of how much they will be interested in its careful perusal, no description being capable of affording any adequate estimate of its interest and value. He says: Prof. Owen declares himself the champion of spontaneous generation, and maintains also that the formation of living beings out of *inanimate matter* by the conversion of physical and chemical into vital modes of force, is a matter of daily and hourly occurrence. Those of our readers interested in the exciting and wonderful problems of the present day, will not fail to obtain this most suggestive discussion upon all the important points involved.

